# Testicular Morphology and Spermatogenesis in *Potamotrygon motoro*: Insights Into Reproduction of Freshwater Stingrays

**DOI:** 10.1002/jmor.70113

**Published:** 2026-01-29

**Authors:** Maria Luiza Ribeiro Delgado, Patricia Postingel Quirino, Luciane Gomes‐Silva, Viktoriya Dzyuba, Borys Dzyuba, Alexandre Ninhaus‐Silveira, Rosicleire Veríssimo‐Silveira

**Affiliations:** ^1^ L.I.NEO ‐ Laboratory of Neotropical Ichthyology UNESP ‐ Universidade Estadual Paulista “Júlio de Mesquita Filho”, Câmpus de Ilha Solteira Ilha Solteira São Paulo Brasil; ^2^ Instituto de Biociências de Botucatu Programa de Pós‐Graduação em Ciências Biológicas (Zoologia) Botucatu São Paulo Brasil; ^3^ South Bohemian Research Center of Aquaculture and Biodiversity of Hydrocenoses, Research Institute of Fish Culture and Hydrobiology, Faculty of Fisheries and Protection of Waters University of South Bohemia in České Budějovice České Budějovice Czech Republic

**Keywords:** helical head, nucleus, sertoli cell, spermatoblast, spermatozoa

## Abstract

This study investigated the spermatogenesis of the freshwater stingray *Potamotrygon motoro* using microscopic analyses. The testes of this species were described as being composed of germinal zones, a degenerated zone, and the epigonal organ, consisting of connective tissue and lymphomyeloid cells. A cystic pattern of spermatogenesis was observed, in which each cyst is formed and maintained by Sertoli cells that undergo morphological and positional changes throughout the process. After the release of spermatozoa into the duct, the peripheral cysts formed a degenerating layer. Spermiogenesis, the final phase of sperm development and differentiation, was identified as a complex process in *P. motoro*, involving nuclear compaction and structural modifications. This study presents, for the first time, a detailed description of the germ cell development process in *P. motoro*, contributing to the understanding of spermatogenesis in freshwater stingrays.

## Graphical abstract

Electron microscopy of Potamotrygon motoro spermatozoa. a – Illustration of a schematic sperm cell depicting the helical head, intermediate piece (midpiece), and flagellum. b ‐ Helical heads (Hh) of spermatozoa inserted into the cytoplasm of Sertoli cells (S) through openings in the plasma membrane. Scales: 10 μm.

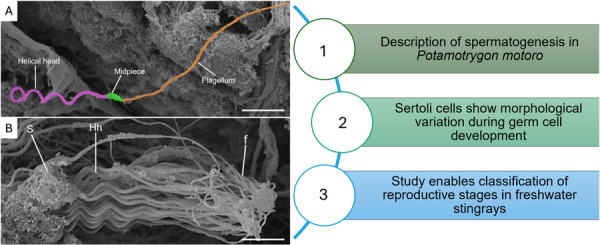

## 1 Introduction

The family Potamotrygonidae belongs to the order Myliobatiformes and is exclusively composed of freshwater stingrays found in South America. This family comprises five genera, one of which is *Potamotrygon* (Fontenelle and de Carvalho 2017; Myers et al. 2025). This genus includes the species *Potamotrygon motoro* (Müller and Henle 1841), which is widely distributed in the Amazon and Paraná–Paraguay river basins, as well as in countries such as Argentina, Bolivia, and Paraguay (Loboda et al. 2016; Nelson et al. 2016; Rizo‐Fuentes et al. 2021).

In recent decades, *P. motoro* has been recorded in the Upper Paraná River region, and the presence of navigation locks in hydroelectric power plants along the Tietê–Paraná river system has facilitated its colonization. Additionally, dams have promoted the expansion of the species into areas where it was not originally part of the native aquatic fauna (Garrone‐Neto et al. 2007; Garrone‐Neto and Haddad Junior 2010; Loboda and Carvalho 2013).

In the northern region of Brazil, from December to February, during the high‐water period of the hydrological cycle, *P. motoro* exhibits a well‐defined reproductive period that is closely linked to the hydrological regime of aquatic environments (Thorson et al. 1983; Charvet‐Almeida et al. 2005). The reproductive cycle is annual, as in most species, and is correlated with the female cycle, ensuring that adequate sperm reserves are available for mating at the onset of ovulation (Yoshida and Asturiano 2020; Marcon et al. 2021).

In *P. motoro*, as in other stingrays, fertilization occurs internally, and females are viviparous, that is, eggs are retained within the female, and offspring are delivered at a more advanced stage of development (Nelson et al. 2016). Males possess claspers, which function as copulatory organs and represent one of the most prominent sexually dimorphic features of stingray species (Carrier et al. 2004).

For some ray species, no differences are observed between the testes on either side, which are considered compound organs. Testes on both sides appear to be developed and presumably functional (Thorson et al. 1983), similar to marine species, in which both testes are functional despite the left side being larger than the right (Soto‐López et al. 2021). In *P. iwamae*, a freshwater species, the right and left testes undergo similar developmental processes, demonstrating the functionality of both organs in this species (Charvet‐Almeida et al. 2005). However, some species may exhibit morphometric differences between their testes, as observed in the butterfly ray *Gymnura marmorata*, in which only the left testis is functional, resulting in testicular asymmetry (Burgos‐Vázquez et al. 2019).

At least three distinct types of testicular organization have been described: the radial type, observed in some shark species (Selachii), in which the most immature germ cells are located in a central germinal zone and spermatocysts mature centrifugally toward the periphery, where spermatozoa are released into the efferent ducts; the diametral type, characterized by a single germinal zone located at one end of the testis, with developing cells maturing across the diameter of the organ toward the opposite end, where spermatozoa are released; and the compound type, defined by the presence of multiple lobules or germinal zones, each with independent spermatogenic activity, resulting in a more complex and spatially heterogeneous testicular organization (Pratt 1988). The compound type occurs in species of the orders Rajiformes and Myliobatiformes, as previously described for species of the genus *Potamotrygon*. It is characterized as a mixture of the other two types, in which spermatocysts formed in germinal zones on the dorsal surface radiate in columns through the testis. The duct is positioned opposite the germinal zones containing the follicles (Del Mar Pedreros‐Sierra and Ramírez‐Pinilla 2015).

In Chondrichthyes, spermatogenesis occurs in the germinal zone, with a spermatogonium and a single Sertoli cell forming a spermatocyst (Moya et al. 2015; Coetzee et al. 2021). This process can be defined as the formation and development of male germ cells, beginning with spermatogonia as the initial cells, progressing through the spermatocyte and spermatid stages, and ultimately forming spermatozoa (Coetzee et al. 2021). Importantly, this process occurs independently of morphological differences in testicular organization, indicating a common developmental pattern across different testicular types (Grier 1992; Yoshida and Asturiano 2020).

Considering the peculiarities and importance of this organ, the objective of this study was to characterize spermatogenesis in this species through histological analysis of the germinal epithelium and the cells present within it. This analysis provides information that supports reproductive and ecological studies of the species, particularly in the context of its recent colonization of new territories.

## 2 Materials and Methods

### 2.1 Obtaining the Animals and Ethical Statement

Ten adult male *P. motoro* individuals were collected with the assistance of local fishermen using harpoons in the Jupiá Reservoir, Paraná River, in the municipality of Ilha Solteira, São Paulo State, Brazil (20°25′52″S, 51°20′17″W). Collections were conducted in February, corresponding to the rainy season in the study area. Sampling was approved by the Biodiversity Authorization and Information System (SISBIO; permit no. 58102‐1). The specimens had a mean total length of 46.6 ± 6.46 cm and a mean disc length of 26.8 ± 3.27 cm and were considered adults. Specimens were anesthetized with an MS‐222 solution, and all procedures were conducted in accordance with the protocol approved by the Animal Ethics and Use Committee (CEUA‐FEIS/UNESP 14/2018).

### 2.2 Sampling of Testes and Microscopy

Testicles from all sampled individuals were collected, and both testes from each animal were used for histological analyses. After removal, the testes were fragmented and fixed in a solution containing 4% paraformaldehyde and 2% glutaraldehyde in Sorensen phosphate buffer (0.1 mol L⁻¹, pH: 7.2) for at least 24 h. The material was then gradually dehydrated through an alcohol series and embedded in historesin (glycol methacrylate) following standard protocols for light microscopy (Zeiss Scope A1 + AxioCam MRc5). Each testis was sectioned into three distinct regions (anterior, middle, and posterior portions of the organ), yielding approximately 10 slides per individual, each containing about eight histological sections of 3 µm thickness. Sections were obtained using a semiautomatic microtome (Leica RM2245) and stained with hematoxylin and eosin, toluidine blue + borax (pH: 7), metanil yellow + ferric hematoxylin, and periodic acid–Schiff (PAS).

Testicular fragments were prepared for analysis by scanning electron microscopy (SEM). Samples were fixed in 2.5% glutaraldehyde, dehydrated through a graded ethanol series, dried using liquid CO₂ in a critical point dryer, and mounted on aluminum stubs. The samples were subsequently sputter‐coated with gold–palladium and examined and photographed using a scanning electron microscope.

## 3 Results

### 3.1 Testicular Morphology

**Figure 1:**
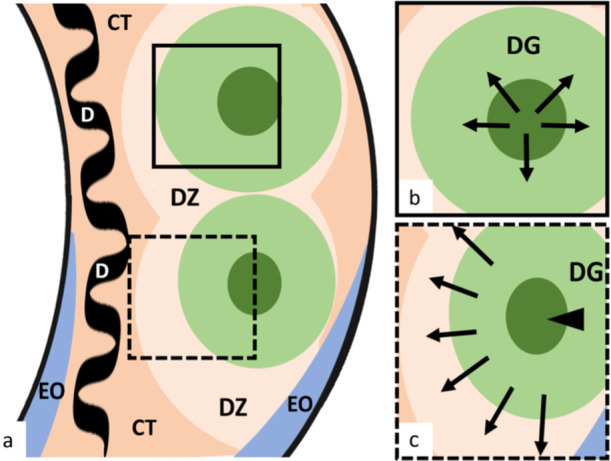
Diagram of the internal morphology and direction of germ cell lineage development in *Potamotrygon motoro*. (A) General view of the internal testicular structure. (B) Higher magnification of the region delimited by the continuous line. (C) Higher magnification of the region delimited by the dashed line. Arrows indicate the radial direction of germ cell development within the Germinal Zone, starting from the Initial Germinal Zone (arrowhead) toward the Developed Germinal Zone (DG) (B), and subsequently entering the Degenerated Zone (DZ) (C). CT, connective tissue; D, duct; EO, epigonal organ.

**Figure 2:**
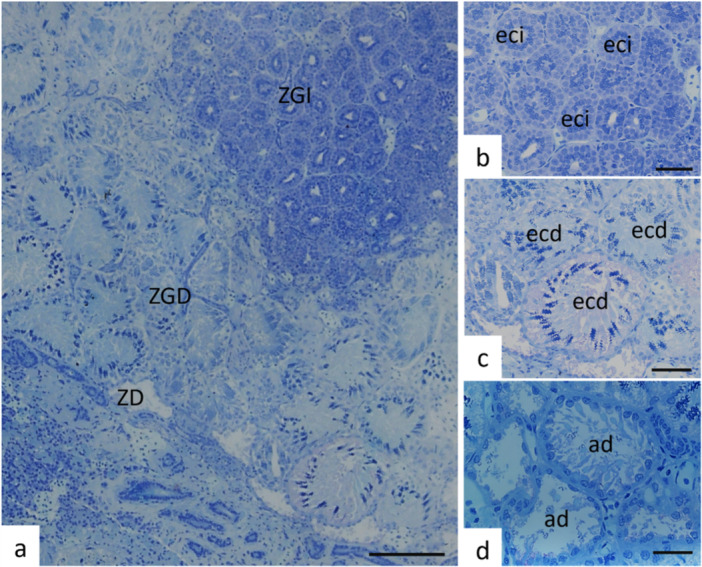
Internal structure of germ cell development in *Potamotrygon motoro*. (a) Delimitation of Zones: Initial Germinal Zone (ZGI), Developed Germinal Zone (ZGD), and Degenerated Zone (ZD). (b) Early developing spermatocysts (eci) present in the initial germinal zone. (c) Advanced developing spermatocysts (ecd) present in the developed germinal zone. (d) Degenerated ampullae (ad) that have already housed spermatids and released them into the lumen of the ducts, present in the degenerated zone. Staining: Toluidine blue, pH: 7.0. Scales: a—50 μm, b, c and d—20 μm.

In *P. motoro*, the testes are paired, cylindrical organs divided into distinct regions (Figure 1). The dorsal portion of the testis consists of germinal zones, where spermatogenesis occurs. The germinal zone is consistently surrounded by a degenerated zone (Figures 1A, and 2A,D) and is associated with the spermatic duct. The epigonal organ, a solid structure composed of connective tissue and lymphomyeloid cells, is located in the ventral portion of each testis.

In the germinal zone, spermatocysts are arranged according to the developmental stages of germ cells; thus, from the periphery toward the center (Figures 1A and 2A), the entire process of spermatogenesis—spermatogonia, spermatocytes, spermatids, and spermatozoa—occurs, characterizing a radial pattern of development. Accordingly, spermatocysts containing cells at early stages of development (Figure 2A,B) are found at the center of the germinal zone, whereas spermatocysts with more advanced germ cells (Figure 2A,C) are located at the periphery of the germinal zone. At the end of spermatogenesis, spermatozoa are released into the efferent ducts, giving rise to the degenerated zone (Figure 2D).

**Figure 3:**
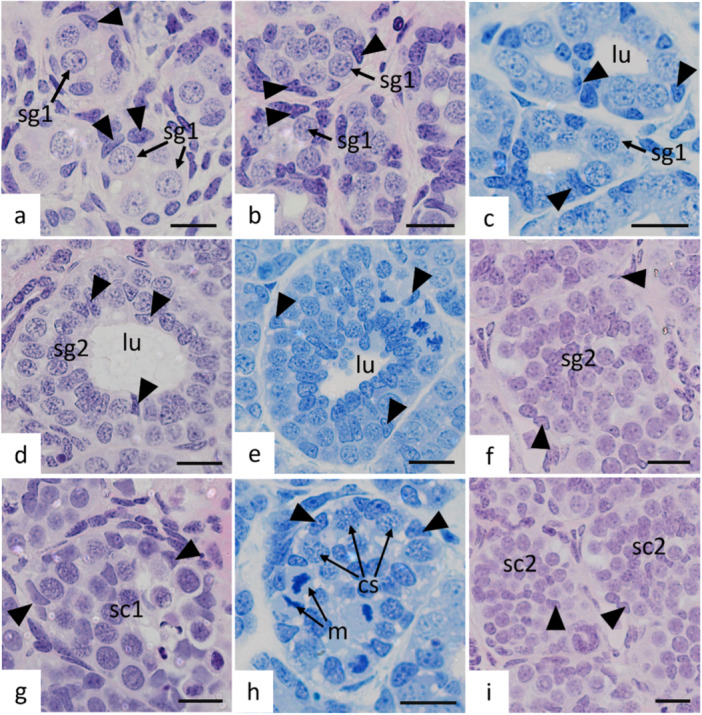
Germ cells in early development of *Potamotrygon motoro*. (a) Spermatogonia 1 (sg1) in cysts of two or more cells surrounded by Sertoli cells (arrowhead). (b) Spermatogonia 1 (sg1) in large cysts after the first cell proliferation. (c) Single‐layer cysts of spermatogonia 1 (sg1) and Sertoli cells with the formation of spermatocystic lumen (lu). (d) Cyst of spermatogonia 2 (sg2) in a two‐layered cyst with the presence of lumen (lu) and Sertoli cells. (e) Cysts of sg2 with reduced lumen (lu). (f) Cyst of sg2 after proliferation, without lumen. g ‐ Spermatocyst of spermatocytes 1 (sc1). (h) Spermatocyst of sc1 with the presence of metaphasic figures (m) and spermatocytes in pachytene (cs). (i) Spermatocysts of spermatocytes 2 (sc2). Staining: a, b, d, f, h, and i—Hematoxylin and Eosin, c, e, and g—Toluidine blue, pH: 7.0. Scales**:** 10 μm.

The primary spermatogonia (Figure 3) of *P. motoro* are the initial and largest cells of the germinal lineage. They possess large nuclei with morphologies ranging from round to oval, loosely condensed chromatin, well‐defined nucleoli, and abundant cytoplasm containing vacuoles. They can be observed either singly or in groups of two or more cells associated with Sertoli cells (Figure 3A–C). Sertoli cells exhibit distinctly comma‐shaped nuclei and intense basophilia.

As spermatogonia undergo successive mitotic divisions, cysts containing daughter cells are formed. Although these cells share many characteristics with their predecessors, they are smaller, have a centrally located nucleus, and are organized into well‐defined cysts composed of multiple layers of germ cells associated with Sertoli cells and a lumen (Figure 3D). From this stage onward, mitotic proliferation within the spermatocysts results in more densely packed cysts lacking a lumen (Figure 3E).

Spermatogonia differentiate into primary spermatocytes, which continue to proliferate within the cysts and are arranged in groups of germ cells accompanied by Sertoli cells that are now positioned at the periphery of the spermatocyst (Figure 3G). Primary spermatocytes (Figure 3H) are characterized by chromatin compaction, leading to the formation of the synaptonemal complex. At this stage, metaphasic figures (Figure 3H), formed during the first meiotic division and giving rise to secondary spermatocytes (Figure 3I), can also be observed.

**Figure 4:**
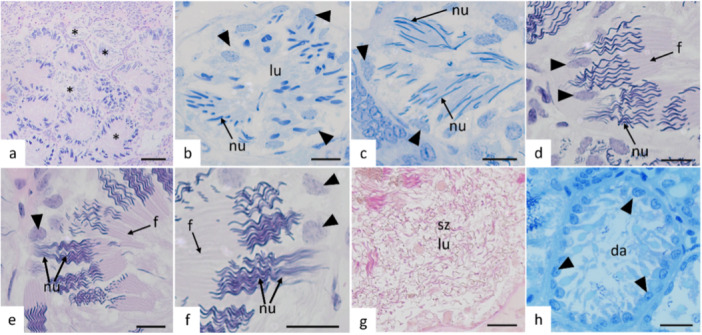
Germ cells of *Potamotrygon motoro* in the stage of spermiogenesis. (a) Peripheral region of the germinal zone showing spermatocysts of spermatids in different stages of differentiation (*). (b) Spermatocyst of spermatids in the early elongation phase, nuclei (nu) shaped like drops turning towards the nuclei of Sertoli cells (arrowhead) located at the periphery, initiating the appearance of the spermatocystic lumen (lu). (c) Elongation and approximation of the nuclei (nu) of spermatids directed towards the nucleus of the Sertoli cell. (d) Spermatids with elongated nuclei (nu) and intermediate coiling, now with well‐defined flagellum (f) formation. (e, f) Advanced coiling of the nuclei (nu) of joined spermatids directed towards the nucleus of the Sertoli cell. (g) Fully formed spermatozoa (sz) released inside the lumen (lu) of the spermatocyst. (h) Degenerated ampulla (da) after the release of germ cells, highlighting Sertoli cells at the periphery of the now disorganized spermatocyst. Flagellum (f). Staining: a, d, e, and f ‐ Hematoxylin and Eosin, b, c, and h ‐ Toluidine blue, pH: 7.0, g ‐ Metanil Yellow + PAS. Scales: a—50 μm, b–h—10 μm.

The spermatids, resulting from the second meiotic division of spermatocytes, undergo a process of differentiation (Figure 4A). This process occurs concurrently with the movement of the spermatid nucleus toward the nucleus of Sertoli cells located at the periphery of the spermatocysts. This realignment causes the spermatocysts to once again contain a lumen (Figure 4B). From this stage onward, elongation of the spermatid nucleus begins. Initially, the nuclei are drop‐shaped and gradually elongate (Figures 4B,C).

**Figure 5:**
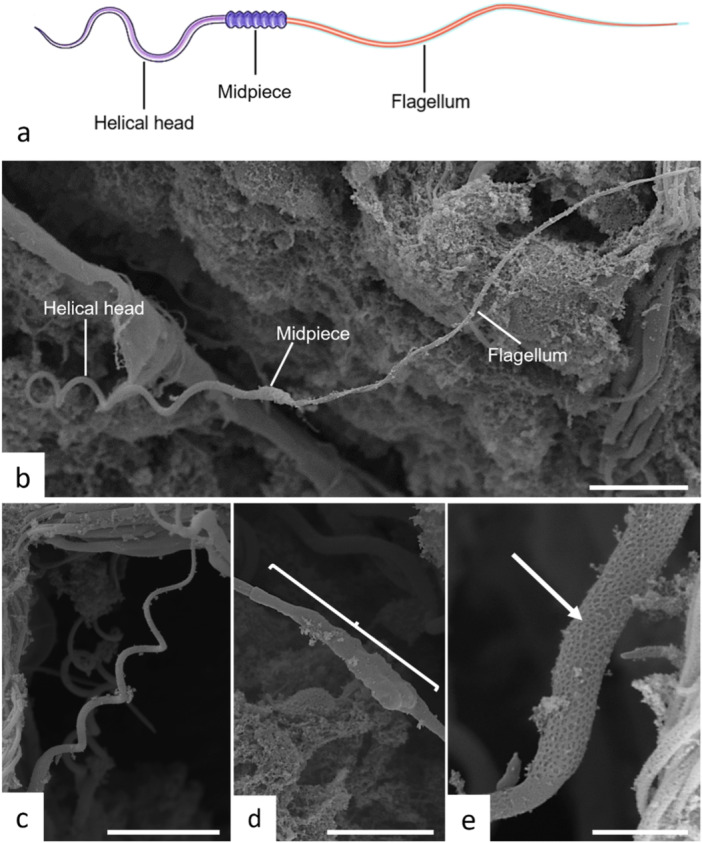
Electron microscopy of *Potamotrygon motoro* spermatozoa. (a and b) Illustration of a schematic sperm cell depicting the helical head, intermediate piece (midpiece), and flagellum. (c) Head structure and nucleus coiling. (d and e) Enlargement of the intermediate piece region (brackets) demonstrating the external porous texture (arrow). Scales: b and c—10 μm, d—5 μm, e—2 μm.

During spermiogenesis and nuclear coiling, spermatids become progressively grouped and continue to move closer to one another. The heads of the spermatids remain oriented toward the nucleus of the Sertoli cell located at the periphery of the cyst, concomitant with gradual coiling and tail formation (Figures 4D–F and 5A–C). During spermiogenesis, as part of the differentiation process, formation of the midpiece also occurs (Figure 5A,B,D,E).

At the end of spermiogenesis, the spermatids become dispersed within the cysts and are no longer grouped (Figure 4G). The spermatozoa are subsequently released into the duct, and the spermatocyst is then referred to as a degenerated ampulla (Figure 4H), contributing to the degenerated zone (Figure 2D).

**Figure 6:**
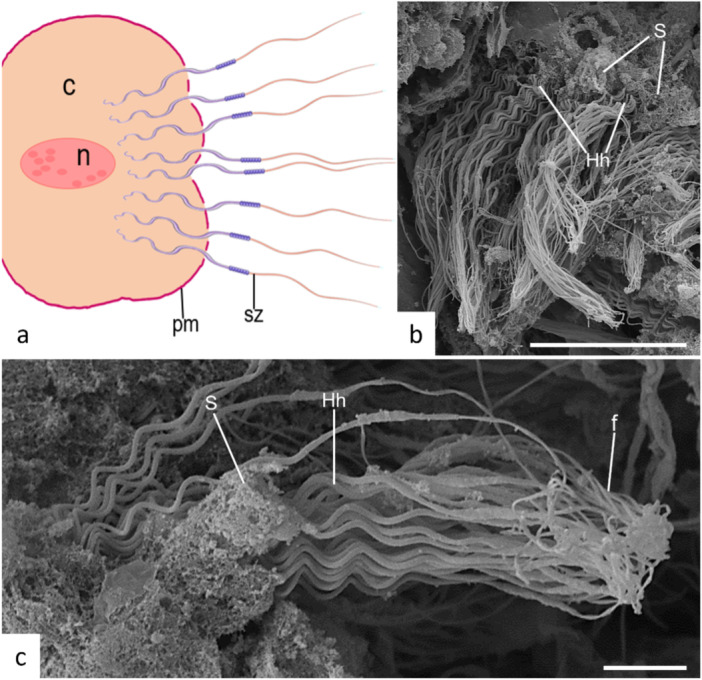
Electron microscopy of *Potamotrygon motoro* spermatoblast. (a) Illustration of a schematic spermatoblast; spermatids (sz), Sertoli cell plasma membrane (pm) and Flagellum (f) are demonstrated. (b and c) Helical heads (Hh) of spermatozoa inserted into the cytoplasm of Sertoli cells (S) through openings in the plasma membrane. Scales: b—50 μm, c—10 μm.

Sertoli cells are present and play crucial roles in the various structures formed within the germinal tissue. One of these structures is the spermatoblast, a group of Sertoli cells and germline cells that originate from a single spermatogonium and are at the same developmental stage. Spermatoblasts are substructures of a spermatocyst, the functional unit of spermatogenesis. At the spermatid stage of spermatogenesis, spermatid heads within spermatoblasts are oriented toward the nuclei of Sertoli cells, whereas their tails project into the lumen of the spermatocysts containing these spermatoblasts (Figure 6A–C).

Sertoli cells undergo several modifications during germ cell development. Nuclear shape changes from triangular, when juxtaposed with early germ cells, to pavement‐like and comma‐shaped during the formation of spermatoblasts within spermatocysts, and finally to a rounded and clear nuclear morphology, particularly associated with the final stages of germ cell development, such as spermatids and spermatozoa. In these final stages, Sertoli cell nuclei have an average diameter of approximately 5 µm.

## 4 Discussion

The development of the germ cell lineage occurs within numerous functional units known as spermatocysts, a well‐defined characteristic previously described in Chondrichthyes species (Pratt 1988; Grier 1992; Coetzee et al. 2021).

In the testicular organization, the continuous formation of germinal cysts within the germinal zone leads to the progressive spatial separation of newly formed cysts as they mature. Some authors have identified three distinct types of testicular organization—radial, diametral, and compound—associated with different groups of elasmobranchs (Stanley 1966; Pratt 1988). This classification remains, to date, the framework most commonly used to describe the testes of various Chondrichthyes species (Yoshida and Asturiano 2020).

The radial type is characterized by the formation of germinal cysts in the central germinal zone of the testicular lobe, with developmental progression occurring from the center toward the periphery. The efferent ducts collect maturing spermatozoa as they move outward. This pattern is observed in species of the order Lamniformes, with the shortfin mako shark (*Isurus oxyrinchus*) representing a well‐documented example (Conde‐Moreno and Galván‐Magaña 2006). In contrast, the diametral type, found in sharks of the family Carcharhinidae, such as *Rhizoprionodon porosus* and *R. lalandii*, is characterized by a single germinal zone extending along the entire length of the testis, opposite the epigonal organ. In this arrangement, developing spermatocysts traverse the diameter of the testis to reach the duct (Dzyuba et al. 2019).

The polyspermatocystic testis described in *P. motoro* develops in zones in which germinal development proceeds from the center toward the periphery, exhibiting a radial pattern within the germinal zones and a diametral pattern outside them. This arrangement classifies the testis as the compound type. A similar testicular organization has also been reported for another freshwater stingray of the same genus, *P. magdalenae* (Del Mar Pedreros‐Sierra and Ramírez‐Pinilla 2015).

The development of germ cells, as well as their characteristics and the sequence in which these processes occur during spermatogenesis, varies among species and taxonomic groups, representing an important factor in understanding species reproduction. The presence of immature individuals with primordial germ cells forming in the connective tissue surrounding the epigonal organ, which almost entirely envelops the testis, is characteristic of the so‐called “raia cururu.” This regional common name is applied to Amazonian species of the genus *Potamotrygon* that exhibit this condition during germ cell formation (Zaiden et al. 2010).

In more distantly related ray groups, such as marine species, germ cell maturation also occurs within cysts, similar to that observed in *P. motoro*. In these species, an expansion of the germinal area accompanied by a gradual reduction of the epigonal organ in the testis, associated with sexual maturation and cellular proliferation, has been documented (Acero et al. 2008; Chemello et al. 2024).

During cellular proliferation, additional structures form within the germinal zones, and the formation of the spermatoblast is a feature that has already been reported in other species. In *P. motoro*, this structure appears to follow a consistent pattern among freshwater stingrays; despite its particularities, it exhibits developmental and modification patterns similar to those observed in other representatives of the group. Both the distinct structures and the spatial arrangement of germ cells in relation to Sertoli somatic cells have been documented in other families of Chondrichthyes (Stanley 1983; Zaiden et al. 2010; Moya et al. 2015; Del Mar Pedreros‐Sierra and Ramírez‐Pinilla 2015; Chemello et al. 2024). In marine sharks and rays, cellular assemblies maintained by Sertoli cells are well established and constitute a fundamental component of germ cell development (Girard et al. 2000; Moya et al. 2015; Coetzee et al. 2021), a pattern similar to that observed in *P. motoro*.

Sertoli cells play a crucial role in spermatogenesis by coordinating and controlling germ cell development within a specialized testicular microenvironment in several species (Alves et al. 2013; Hai et al. 2014; Dimitriadis et al. 2015; Coetzee et al. 2021; Heinrich et al. 2021). Owing to their close interaction with gamete development, these cells occur in different configurations and testicular structures, including groups of early germ cells, such as isolated spermatogonia associated with a single Sertoli cell, a configuration previously described (Pratt 1988; Poulakis and Grier 2014). Additionally, configurations are observed within spermatocysts, where a single‐layered ring is formed as a result of their association with spermatogonia (Chatchavalvanich et al. 2005).

In the spermatocyst configuration, Sertoli cells in *P. motoro* migrate from the center of the cyst toward the periphery as germ cells develop. They are already observed in this peripheral position from the formation of primary spermatocytes and remain there until cyst degeneration. This displacement of Sertoli cells has also been reported in other groups of rays, such as species within the order Rajiformes that exhibit this characteristic (Rossouw 2014).

Throughout spermatogenesis in *P. motoro*, Sertoli cells show marked variations in size and shape, affecting both the nucleus and cytoplasm, a pattern similar to that observed in *P. cf. histrix*. In the latter species, significant differences in Sertoli cell length, area, and width have been reported in relation to the developmental stage of the associated germ cells (Zaiden et al. 2010). The largest cell area is associated with stages involving spermatids and spermatozoa. This feature is also evident in *P. motoro*, and such morphological modifications may be related to increased hormonal levels during the reproductive cycle, coinciding with increases in testicular mass, proliferation of spermatocysts, and the expansion of Sertoli somatic cells associated with steroid production (Morales‐Gamba et al. 2019). These factors are consistent with the role of Sertoli cells in coordinating and regulating germ cell development (Hai et al. 2014).

Leydig cells, which are occasionally observed in the germinal epithelium of elasmobranchs, were not identified in *P. motoro*. This absence warrants consideration, as it has not been reported for many species and reinforces the role of Sertoli cells as the primary regulators of germ cell development and potential sources of steroidogenesis in this species (Engel and Callard 2005). Although Leydig cells have been described in *P. magdalenae*, and without excluding their possible involvement in spermatogenesis, Sertoli cells remain fundamental owing to their close structural and functional association with germ cells (Del Mar Pedreros‐Sierra and Ramírez‐Pinilla 2015).

During the coordinated cellular proliferation mediated by Sertoli cells, spermatids undergo differentiation processes that culminate in the formation of helically shaped spermatozoa, as previously described for *P. motoro* (Morales‐Gamba et al. 2019). This spiraling process has been documented in several elasmobranch species (Chatchavalvanich et al. 2005; Zaiden et al. 2010; Morales‐Gamba et al. 2019). Rossouw (2014), studying *Rhinobatos annulatus* (Rajiformes), described three distinct stages of sperm differentiation. From stage II onward, spermatids exhibit a vermiform morphology, marking the onset of elongation and spiraling, which progresses gradually through stages III and IV until fully formed spermatozoa are produced.

When combined with microscopic characteristics, macroscopic stages of gonadal maturation provide an effective framework for classifying freshwater stingrays. This approach has been adopted by several authors, who recognize the following phases: immature, maturing, actively mature, and resting mature. This classification is currently applied to species of the family Potamotrygonidae and remains widely used in reproductive studies of freshwater rays (Del Mar Pedreros‐Sierra and Ramírez‐Pinilla 2015; Marcon et al. 2021).

## 5 Conclusion

With the specific characteristics of the germ cells of *Potamotrygon motoro* described here, it becomes possible to effectively classify reproductive stages. The information provided in this study on the reproduction of this species represents the first in‐depth description of germ cell characteristics and serves as a foundation for further reproductive studies.

## Author Contributions

**Maria Luiza Ribeiro Delgado:** sample collection, conceptualizations, methodology, formal analysis, data curation, writing – original draft. **Patricia Postingel Quirino:** conceptualizations, methodology, reviewing. **Luciane Gomes‐Silva:** sample collection, reviewing. **Viktoriya Dzyuba:** sample collection, reviewing. **Borys Dzyuba:** sample collection, reviewing. **Alexandre Ninhaus‐Silveira:** conceptualizations, reviewing. **Rosicleire Veríssimo‐Silveira:** conceptualizations, reviewing, editing, supervision.

## Conflicts of Interest

The authors declare no conflicts of interest.

## Data Availability

All data related to this research is available in the university's public repository, at the following website: https://repositorio.unesp.br/entities/publication/fb1d1fac-3f38-4343-a270-a8b4d756ec95.
